# Aberrant Expression and Oncogenic Activity of SPP1 in Hodgkin Lymphoma

**DOI:** 10.3390/biomedicines13030735

**Published:** 2025-03-17

**Authors:** Stefan Nagel, Corinna Meyer

**Affiliations:** Human and Animal Cell Lines, Leibniz-Institute DSMZ, 38124 Braunschweig, Germany

**Keywords:** DLBCL, homeobox, homeodomain, MCL, PCNSL

## Abstract

**Background**: Hodgkin lymphoma (HL) is a B-cell-derived malignancy and one of the most frequent types of lymphoma. The tumour cells typically exhibit multiple genomic alterations together with aberrantly activated signalling pathways, driven by paracrine and/or autocrine modes. SPP1 (alias osteopontin) is a cytokine acting as a signalling activator and has been connected with relapse in HL patients. To understand its pathogenic role, here, we investigated the mechanisms and function of deregulated SPP1 in HL. **Methods**: We screened public patient datasets and cell lines for aberrant SPP1 expression. HL cell lines were stimulated with SPP1 and subjected to siRNA-mediated knockdown. Gene and protein activities were analyzed by RQ-PCR, ELISA, Western blot, and immuno-cytology. **Results**: *SPP1* expression was detected in 8.3% of classic HL patients and in HL cell line SUP-HD1, chosen to serve as an experimental model. The gene encoding *SPP1* is located at chromosomal position 4q22 and is genomically amplified in SUP-HD1. Transcription factor binding site analysis revealed TALE and HOX factors as potential regulators. Consistent with this finding, we showed that aberrantly expressed PBX1 and HOXB9 mediate the transcriptional activation of *SPP1*. RNA-seq data and knockdown experiments indicated that SPP1 signals via integrin ITGB1 in SUP-HD1. Accordingly, SPP1 activated NFkB in addition to MAPK/ERK which in turn mediated the nuclear import of ETS2, activating oncogenic *JUNB* expression. **Conclusions**: *SPP1* is aberrantly activated in HL cell line SUP-HD1 via genomic copy number gain and by homeodomain transcription factors PBX1 and HOXB9. SPP1-activated NFkB and MAPK merit further investigation as potential therapeutic targets in affected HL patients.

## 1. Introduction

Hodgkin lymphoma (HL) is one of the most frequent B-cell-derived lymphomas, displaying peak incidences around ages 20 and 60 years [1]. Two main histopathologic subtypes are distinguished: classic HL (cHL) and nodular lymphocyte predominant HL (NLPHL) [2]. HL patients may be effectively treated by chemotherapy and in certain cases by radiotherapy, mostly combined with a good prognosis. However, relapsing HL threatens unfavourable outcomes, demanding alternative treatment protocols [3].

Hallmarks of HL tumour cells include aberrantly activated pathways, like NFkB-signalling, JAK-STAT- and MAPK/ERK-pathways [4]. Furthermore, cHL tumour cells show deregulations of specific transcription factors (TFs), including aberrantly activated NFkB factors and AP-1 factor JUNB [1,5], in addition to the aberrant downregulation of B-cell-specific TFs, including basic helix-loop-helix factor TCF3 and homeodomain factor PAX5 [1]. Homeodomain (HD)-containing TFs are generally involved in differentiation processes, and in carcinogenesis when deregulated [6,7]. These factors are classified according to HD sequence similarities [8]. Accordingly, TALE-class HD TFs possess a three-amino-acid-long HD extension and cooperate with HD factors of the HOX family [9]. Finally, several members of the ETS-family of TFs have also been described to be deregulated in HL [10].

HL tumour cells are called Hodgkin and Reed–Sternberg (HRS) cells in cHL and LP cells in NLPHL [1]. Both are surrounded by non-malignant activated immune cells, and thus represent a mere fraction of the tumour mass. This feature is related to pathological impacts of aberrant signalling activities, driven by external and internal factors. According to the ligand’s source, signalling pathways may be activated in either paracrine or autocrine modes. Cytokines may act as ligands and initiate numerous activities in normal and malignant immune cells [11]. In HL, several cytokines play a pathogenic role, notably IL6, IL7, IL13 and IL21 [1,4]. The cytokine SPP1 (secreted phosphoprotein 1, alias osteopontin) is about 300-amino-acids long, and differs due to alternative splicing. SPP1 interacts with components of the extracellular matrix, and with CD44 and integrins, forming common receptors for this ligand [12,13]. In HL, *SPP1* activity has been connected with relapse, thus representing a biomarker which may directly contribute to a worsened prognosis, demanding a bespoke therapy [3].

Authentic, well-characterized hematopoietic malignant cell lines comprise a resource for discovering oncogenomic changes and the preclinical testing of targeted therapies [14]. To study HL and investigate pathogenic mechanisms in this unique type of cancer, several bona fide HL cell lines have been established, combining thorough characterization and faithful disease modellings [15]. Here, we investigated the regulation and function of aberrantly expressed signalling factor SPP1 in HL by both analyzing patient datasets from different types of B-cell lymphoma and by exploiting HL cell lines as experimental models.

## 2. Materials and Methods

### 2.1. Bioinformatic Analyses

Expression data from HL, diffuse large B-cell lymphoma (DLBCL), and mantle cell lymphoma (MCL) patients were obtained from the Gene Expression Omnibus (GEO, www.ncbi.nlm.nih.gov). We used gene expression profiling datasets GSE12453 and GSE16455 [16,17,18]. For the screening of transcriptional activity in cell lines, we analyzed RNA-sequencing data from 100 leukemia/lymphoma cell lines which are available at ArrayExpress (www.ebi.ac.uk/arrayexpress, accessed on 20 December 2024) via E-MTAB-7721 [19]. These RNA-seq data were visualized using the online tool DSMZCellDive (https://celldive.dsmz.de/) [20]. Gene expression analysis of selected tissues and hematopoietic cell types was performed using The Human Protein Atlas (www.proteinatlas.org) [21]. Identification of potential transcription factor (TF) binding sites was performed using the UCSC genome browser (https://genome.ucsc.edu).

### 2.2. Cell Lines and Treatments

HL cell lines are held by the DSMZ (Braunschweig, Germany), cultivated as de-scribed (www.dsmz.de), and were authenticated and tested negative for mycoplasma infection. Transcriptional suppression was performed by treatment with gene specific siRNA oligonucleotides in comparison to AllStars negative Control siRNA (siCTR) obtained from Qiagen (Hilden, Germany). All used siRNAs are listed in Table S1. For overexpression assays, we used expression construct pCMV6-ETS2 (Origene, Herford, Germany). SiRNAs (80 pmol) and plasmid DNA (2 µg) were transfected into 1 × 10^6^ cells by electroporation using the EPI-2500 impulse generator (Fischer, Heidelberg, Germany) at 350 V for 10 ms. Electroporated cells were harvested after 20 h of cultivation. Cell lines were additionally treated with 14 µM of NFkB-inhibitor (NF-kB Activation Inhibitor IV, #481412), or 25 µM of ERK-inhibitor PD98059, with both dissolved in DMSO and obtained from Sigma-Aldrich (Taufkirchen, Germany), with 20 ng/mL of SPP1 protein (Bio-Techne, Wiesbaden, Germany), and harvested after 20 h cultivation.

Functional testing was performed using the IncuCyte S3 Live-Cell Imaging Analysis System (Sartorius, Göttingen, Germany). Apoptotic cells were detected using the IncuCyte Caspase-3/7 Green Apoptosis Assay diluted at 1:2000 (Sartorius). Data analysis was performed using the Cell-by-Cell software tool (Sartorius, Incucyte 2022B Rev2). Live-cell imaging experiments were performed twice with fourfold parallel tests.

### 2.3. Polymerase Chain Reaction (PCR) Analyses

Total RNA was extracted from cultivated cell lines using TRIzol reagent (Invitrogen, Darmstadt, Germany) or the RNeasy Plus extraction kit (Qiagen). Primary human total RNA was purchased from Biochain/BioCat (Heidelberg, Germany). cDNA was synthesized using 1 µg of RNA, random priming and Superscript II (ThermoFisher Scientific, Darmstadt, Germany). Real-time quantitative (RQ)-PCR analysis was performed using the 7500 Real-time System and commercial buffer and primer sets (ThermoFisher Scientific). For the normalization of expression levels, we quantified the transcripts of the TATA box binding protein (TBP). For the quantification of SPP1 genomic copy numbers, we used the following oligonucleotides which were obtained from Eurofins MWG (Ebersberg, Germany): SPP1-for 5′-TTGCCCAGGACCTG-3′ and SPP1-rev 5′-GCTCATTGCTCTCATCATTGGC-3′. The locus of MEF2C was used as a control: MEF2C-for 5′-GCAGGAATTTGGGAACTGAG-3′ and MEF2C-rev 5′-CCCATAGTCCCCGTTTTTCT-3′. Quantitative analyses were performed as biological replicates and measured in triplicate. Standard deviations are presented in the figures as error bars. Statistical significance was assessed by Student’s *t*-Test (two-tailed) and the calculated *p*-values are indicated by asterisks (* *p* < 0.05, ** *p* < 0.01, *** *p* < 0.001) or as not significant (n.s.).

### 2.4. Protein Analysis

In cell line supernatants, SPP1 protein was quantified by Enzyme-Linked Immunosorbant Assay (ELISA) using the Human Osteopontin (OPN) Quantikine ELISA Kit (Bio-Techne, #DOST00). Supernatants were harvested from 1 × 10^6^ cells growing in 1 mL of medium for 24 h and stored at −20 °C.

Western blots were performed by the semi-dry method. Cell line protein lysates were prepared using the SIGMAFast protease inhibitor cocktail (Sigma-Aldrich). Extracted proteins were separated in SDS-gels and transferred onto nitrocellulose membranes (Bio-Rad, München, Germany) and blocked with 5% dry milk powder dissolved in a phosphate-buffered-saline buffer (PBS). We used the following antibodies: alpha-Tubulin (Sigma-Aldrich, #T6199) and phospho-ERK (Santa Cruz Biotechnology, Heidelberg, Germany #sc-7383). For loading control, blots were reversibly stained with Poinceau (Sigma-Aldrich) and the detection of alpha-Tubulin (TUBA) was performed thereafter. Secondary antibodies were linked to peroxidase for detection by Western-Lightning-ECL (Perkin Elmer, Waltham, MA, USA). For documentation, we used the digital system ChemoStar Imager (INTAS, Göttingen, Germany).

For protein detection within fixed cells, we performed immuno-cytology as follows: Cells were spun onto slides, air-dried and fixed with methanol/acetic acid for 90 s. The polyclonal ETS2 antibody was derived from rabbit (Biozol, Eching, Germany #GTX104527), diluted at a ratio of 1:20 in PBS containing 5% BSA, and incubated for 30 min. The slides were subsequently washed 3 times with PBS. Preparations were then incubated with secondary FITC-labelled anti-rabbit IgG-antibody (Santa Cruz Biotechnology, #sc-2359) which was diluted at a ratio of 1:100 in PBS containing 5% BSA and incubated for 20 min. After the final washing, the cells were mounted in Vectashield (Vector Laboratories, Burlingame, CA, USA), containing 4′,6-diamidino-2-phenylindol (DAPI) for nuclear staining. Images were captured with an Axion A1 microscope using Axiocam 208 colour and software ZEN 3.3 blue edition (Zeiss, Göttingen, Germany).

### 2.5. Genomic Profiling Analysis

For genomic profiling, genomic cell line DNA was prepared by the Qiagen Gentra Puregene Kit (Qiagen, Hilden, Germany). Labelling, hybridization and scanning of Cytoscan HD arrays was processed by the Genome Analytics Facility located at the Helmholtz Centre for Infection Research, according to the manufacturer’s protocols (Affymetrix, High Wycombe, UK). Data analysis was performed using the Chromosome Analysis Suite software version 3.1.0.15 (Affymetrix) and copy number alterations were determined accordingly.

## 3. Results

### 3.1. SPP1 Expression in HL Patients and Cell Lines

**Figure 1:**
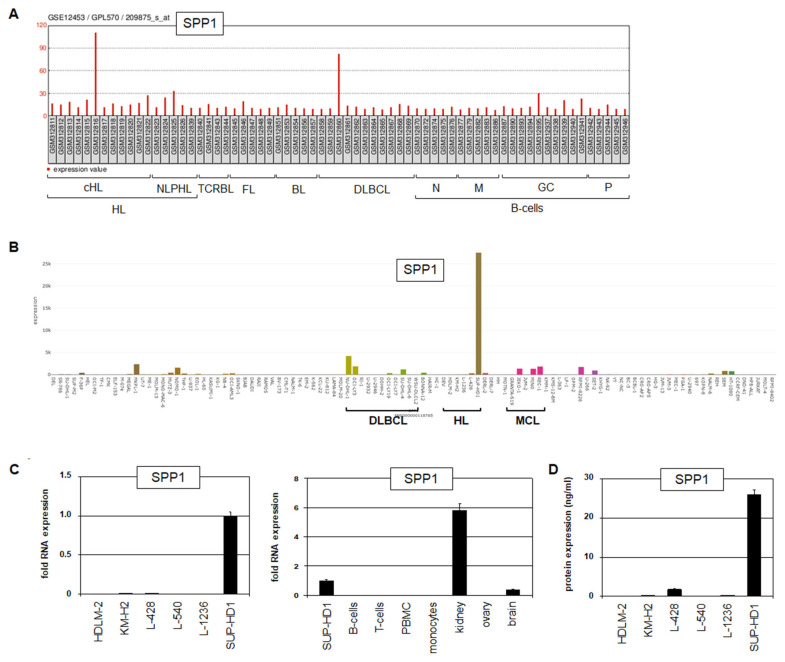
Expression of SPP1 in HL. (**A**) Analysis of public gene expression profiling dataset GSE12453 shows aberrant SPP1 activity in subsets of HL and DLBCL patients while B-cells tested negative. The y-axis indicates expression levels, the x-axis indicates the patient samples. Abbreviations: BL: Burkitt lymphoma; cHL: classic HL; DLBCL: diffuse large B-cell lymphoma; FL: follicular lymphoma; GC: germinal centre; M: memory B-cells; N: naïve B-cells; NLPHL: nodular lymphocyte predominant HL; P: plasma cell; TCRBL: T-cell-rich B-cell lymphoma. (**B**) RNA-seq data for SPP1 in 100 leukemia/lymphoma cell lines. Indicated are DLBCL (NU-DHL-1, OCI-LY3, RI-1, U-2932, U-2946, DOHH-2, OCI-LY19, OCI-LY7, SU-DHL-4, SU-DHL-6, and WSU-DLCL2), HL (DEV, HDLM-2, KM-H2, L-1236, and L-428, SUP-HD1) and mantle cell lymphoma (MCL, cell lines GRANTA-519, JEKO-1, JVM-2, MINO, and REC-1). (**C**) RQ-PCR analysis of *SPP1* in HL cell lines (left) and primary cells including peripheral blood mononuclear cells (PBMCs) and tissues (right). (**D**) ELISA data for SPP1 in supernatants of HL cell lines.

Analysis of public gene expression profiling dataset GSE12453 showed enhanced activity of SPP1 in 1/12 (8.3%) cHL patients, and its absence in NLPHL patients, and mature and developing B-cells including germinal centre B-cells (Figure 1A). In addition, we detected *SPP1* overexpression in 1/11 (9.1%) DLBCL patients (Figure 1A), and in 1/22 (4.5%) MCL patients (Figure S1), showing that *SPP1* upregulation plays a role in subsets of selected B-cell lymphomas. Analysis of *SPP1* transcripts in normal tissues and cell types demonstrated physiological expression in the brain, gallbladder, kidney and placenta (Figure S1B). Basophils, neutrophils and progenitor cells were the only hematopoietic cell types found to express physiological *SPP1* (Figure S1C), further supporting that this gene is silent in B-cells. Thus, *SPP1* is aberrantly expressed in subsets of particular B-cell lymphomas, including HL, DLBCL and MCL.

Here, we focused on *SPP1* in HL, aiming to understand its deregulation and pathogenic role. Screening of RNA-seq data from 100 leukemia/lymphoma cell lines showed elevated expression of SPP1 in HL cell line SUP-HD1, while lower levels were detected in cell lines derived from DLBCL and MCL (Figure 1B), confirming our findings in corresponding patients. RQ-PCR analysis confirmed *SPP1* expression in SUP-HD1, which was higher when compared to primary brain expression but lower than primary kidney expression (Figure 1C). Furthermore, these data show the absence of *SPP1* expression in primary hematopoietic cells, including B-, T-, and peripheral blood mononuclear cells (Figure 1C). SPP1 protein expression was quantified by ELISA in supernatants of six HL cell lines, revealing significant levels (about 26 ng/mL) for SUP-HD1 only (Figure 1D). Taken together, aberrantly expressed *SPP1* was detected in subsets of HL patients and the HL cell line SUP-HD1, endorsing its choice as a model to investigate the regulation and function of SPP1 in this type of B-cell lymphoma.

### 3.2. Aberrant Activation of SPP1 in HL

**Figure 2:**
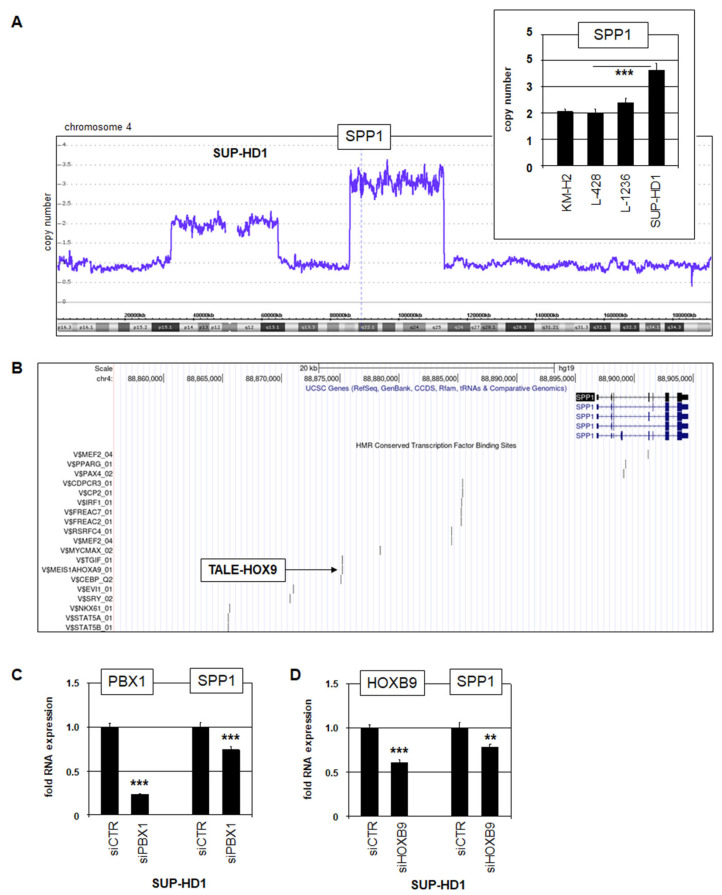
Mechanisms of aberrant *SPP1* activation in HL. (**A**) Genomic profiling data for chromosome 4 from HL cell line SUP-HD1. *SPP1* is located at 4q22 and is part of focal genomic gain. This copy number gain was confirmed by RQ-PCR analysis (insert). (**B**) TF binding site analysis for the gene *SPP1* using the UCSC genome browser. A binding site for TALE-HOX9 factors is indicated. (**C**) RQ-PCR analysis of *PBX1* and *SPP1* after siRNA-mediated knockdown of *PBX1* (left), and of *HOXB9* and *SPP1* after the knockdown of *HOXB9* in SUP-HD1 (right). Statistical significance was assessed by Student’s *t*-Test (two-tailed) and the calculated *p*-values are indicated by asterisks (** *p* < 0.01, *** *p* < 0.001).

In hematopoietic malignancies including HL, genomic rearrangements frequently underlie aberrant gene deregulation [22,23,24,25]. Accordingly, genomic copy number analysis of SUP-HD1 revealed a focal gain for *SPP1* located at chromosomal position 4q22, which was additionally confirmed by RQ-PCR (Figure 2A).

To examine the transcriptional (de)regulation of *SPP1*, we inspected potential TF binding sites using the UCSC genome browser. Intriguingly, the data revealed a binding site for HOX and TALE homeodomain TFs (Figure 2B). Reportedly, HOX-member HOXB9 and TALE-member PBX1 are aberrantly expressed in HL, including SUP-HD1 [9,26]. Consistently, siRNA-mediated knockdown of *PBX1* and *HOXB9* resulted in reduced *SPP1* expression, demonstrating that these factors activate *SPP1* expression in this cell line (Figure 2C). RNA-seq data and genomic profiling data confirmed the aberrant expression of *PBX1* and *HOXB9* in SUP-HD1 while a copy number gain was detectable for *PBX1* only (Figure S2). Thus, *SPP1* is aberrantly activated in HL cell line SUP-HD1 via genomic copy number alteration and overexpressed homeodomain TFs PBX1 and HOXB9.

### 3.3. SPP1-Signalling in HL

**Figure 3:**
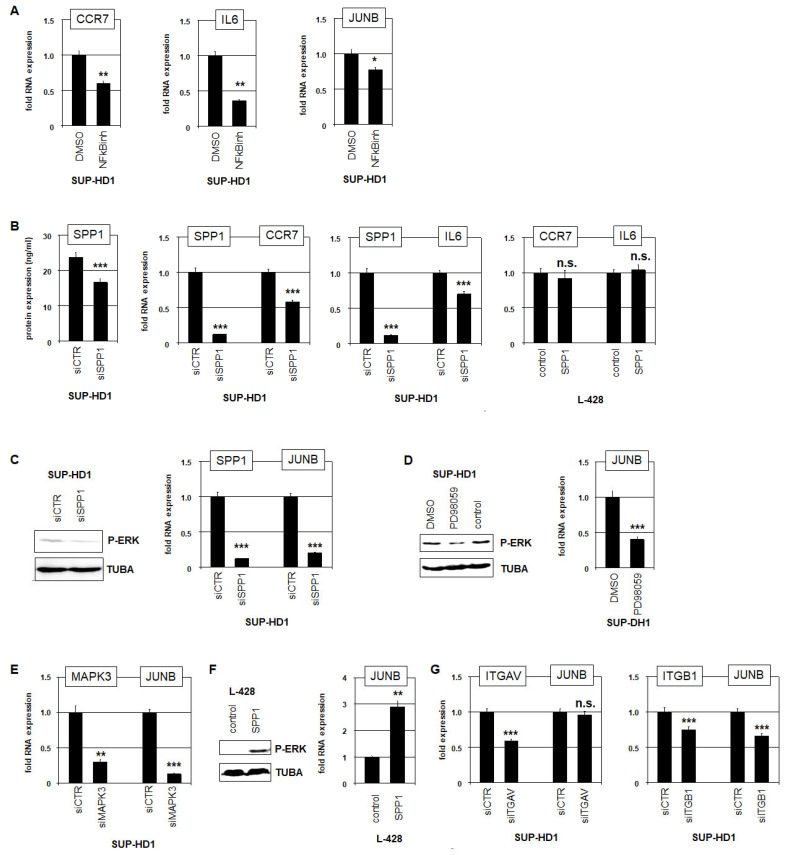
SPP1 activates NFkB, JUNB, and MAPK/ERK in HL. (**A**) RQ-PCR analysis of NFkB-target genes *CCR7*, *IL6* and *JUNB* in SUP-HD1 treated with NFkB-inhibitor. (**B**) ELISA analysis of SPP1 in supernatants of SUP-HD1 cells treated for SPP1-knockdown (left). RQ-PCR analysis of *SPP1* and *CCR7*, and of *SPP1* and *IL6* in SUP-HD1 treated for SPP1-knockdown (middle). RQ-PCR analysis of *SPP1* and *CCR7*, and of *SPP1* and *IL6* in L-428 stimulated with SPP1 (right). (**C**) Western blot analysis of phospho-ERK and TUBA (serving as loading control) of SUP-HD1 treated for SPP1-knockdown (left). RQ-PCR analysis of *SPP1* and *JUNB* in SUP-HD1 treated for SPP1-knockdown (right). (**D**) Western blot analysis of phospho-ERK and TUBA (serving as loading control) of SUP-HD1 treated with ERK-inhibitor PD98059 (left). RQ-PCR analysis of *JUNB* in SUP-HD1 treated with ERK-inhibitor PD98059 (right). (**E**) RQ-PCR analysis of *MAPK3* and *JUNB* in SUP-HD1 treated for MAPK3-knockdown. (**F**) Western blot analysis of phospho-ERK and TUBA of L-428 stimulated with SPP1 (left). RQ-PCR analysis of *JUNB* in L-428 stimulated with SPP1 (right). (**G**) RQ-PCR analysis of *ITGAV*, *ITGB1* and *JUNB* in SUP-HD1 treated for knockdown of *ITGAV* (left) and *ITGB1* (right). Statistical significance was assessed by Student’s *t*-Test (two-tailed) and the calculated *p*-values are indicated by asterisks (* *p* < 0.05, ** *p* < 0.01, *** *p* < 0.001) or as not significant (n.s.).

SPP1 acts as a ligand which interacts with CD44 and/or integrins [12,13]. RNA-seq data showed the downregulation of *CD44* in SUP-HD1 while integrins *ITGAV* and *ITGB1* were highly expressed (Figure S3), indicating that SPP1-signalling operates in this cell line via these integrins. Integrin-mediated SPP1-signalling activates MAPK/ERK, and NFkB and AP1 factors [12,13]. To investigate the role of these downstream activities for SPP1-signalling, we quantified NFkB-targets *CCR7* and *IL6*, and AP1-factor *JUNB*. TF binding site analysis for *CCR7*, *IL6* and *JUNB* confirmed the presence of potential NFkB-sites at these loci (Figures S4 and S5). Accordingly, treatment of SUP-HD1 cells with NFkB-inhibitor resulted in the reduced expression of *CCR7*, *IL6* and *JUNB* (Figure 3A), confirming their regulation by NFkB. Furthermore, siRNA-mediated knockdown of *SPP1* in SUP-HD1, which was confirmed at the protein and RNA-level (Figure 3B), mediated the downregulation of *CCR7* and *IL6* (Figure 3B). Stimulation of SPP1-negative cell line L-428 with SPP1 showed no effect on *CCR7* and *IL6* expression (Figure 3B), suggesting that NFkB-activation may not represent the main signalling effect.

SPP1-knockdown in SUP-HD1 resulted in the reduced phosphorylation of ERK and the downregulation of *JUNB* expression (Figure 3C). Consistently, *JUNB* expression is reportedly also regulated via activated kinases MAPK/ERK [27]. Accordingly, treatment of SUP-HD1 cells with ERK-inhibitor PD98059 resulted in the downregulation of *JUNB* expression (Figure 3D). Moreover, corresponding knockdown experiments showed that MAPK3/ERK1 represents an activator of *JUNB* in SUP-HD1 (Figure 3E). Moreover, stimulation of SPP1-negative HL cell line L-428 with SPP1 protein resulted in the phosphorylation/activation of MAPK/ERK and elevated *JUNB* expression (Figure 3F), highlighting these downstream effects. Finally, we performed the siRNA-mediated knockdown of integrins *ITGAV* and *ITGB1*. Downregulation of *ITGB1* inhibited *JUNB* expression while that of *ITGAV* showed no effect (Figure 3G), demonstrating that SPP1 activates *JUNB* via integrin ITGB1 in HL cell line SUP-HD1. Collectively, our data show that SPP1 controls the activation of NFkB, MAPK/ERK, and *JUNB* expression in HL cells.

### 3.4. ETS2 Activation via SPP1-Signalling

**Figure 4:**
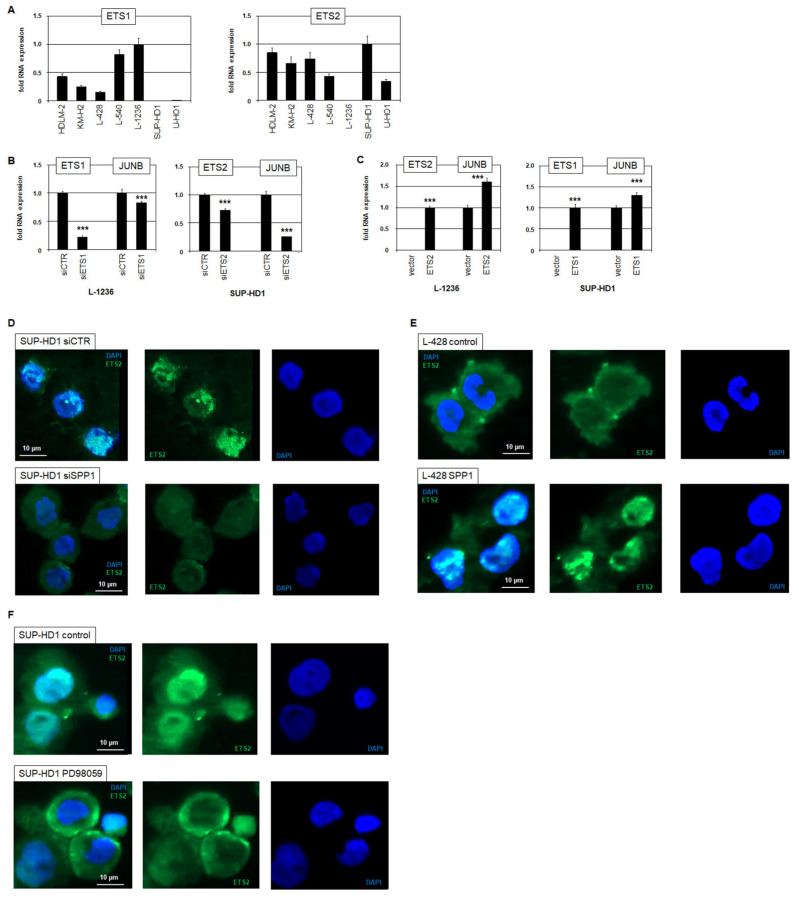
Expression and activity of ETS1 and ETS2 in HL. (**A**) RQ-PCR analysis of *ETS1* (left) and *ETS2* (right) in seven HL cell lines. (**B**) RQ-PCR analysis of *ETS1* and *JUNB* in L-1236 treated for ETS1-knockdown (left). RQ-PCR analysis of *ETS2* and *JUNB* in SUP-HD1 treated for ETS2-knockdown (middle). (**C**) RQ-PCR analysis of *ETS2* and *JUNB* in L-1236 (left), and of *ETS1* and *JUNB* in SUP-HD1 (right), treated for respective forced ETS2 and ETS1 expression. Statistical significance was assessed by Student’s *t*-Test (two-tailed) and the calculated *p*-values are indicated by asterisks (*** *p* < 0.001). (**D**) Immuno-fluorescence microscopical analysis of SUP-HD1 siRNA-control (above) and after siRNA-mediated knockdown of *SPP1* (below). The nuclei of the cells were labelled with DAPI (blue), and ETS2 protein was detected by a primary anti-ETS2 and a FITC-labelled secondary antibody, and is shown in green. (**E**) Immuno-fluorescence microscopical analysis of L-428 controls (above) and after stimulation with SPP1 (below). (**F**) Immuno-fluorescence microscopical analysis of SUP-HD1 controls (above) and after treatment with ERK-inhibitor PD98059 (below). The nuclei of the cells were labelled with DAPI (blue), and ETS2 protein is shown in green.

*JUNB* is aberrantly overexpressed in cHL, representing a key oncogene in this malignancy [5]. Therefore, we analyzed the SPP1-mediated mechanism of *JUNB* activation in HL cell lines. In addition to NFkB-sites, *JUNB* contains a binding site for ETS factors (Figure S5), which are known regulators of this gene [28,29]. *ETS1* and *ETS2* are reportedly deregulated in HL [10], but show contrasting activities in HL cell lines SUP-HD1 and L-1236 (Figure 4A). This particular expression pattern was used to pursue their role in *JUNB* regulation. SiRNA-mediated knockdown of *ETS1* in L-1236 and of *ETS2* in SUP-HD1 resulted in the downregulation of *JUNB*, showing their activating impact (Figure 4B). Furthermore, forced expression of *ETS2* in ETS2-negative L-1236 and of *ETS1* in ETS1-negative SUP-HD1 elevated *JUNB* expression (Figure 4C), confirming that ETS1 and ETS2 act as activators of *JUNB* transcription in HL.

To investigate the relationship between the demonstrated ETS2-mediated regulation of *JUNB* with aberrant SPP1 activity in HL, we performed immuno-cytological assays. Accordingly, siRNA-mediated knockdown of *SPP1* in SUP-HD1 mediated the transfer of ETS2 from the nucleus into the cytoplasm (Figure 4D), while stimulation of SPP1-negative L-428 cells with SPP1 protein mediated the transfer of ETS2 protein from the cytoplasm into the nucleus (Figure 4E). Furthermore, treatment of SUP-HD1 with ERK-inhibitor PD98059 supported the export of ETS2 from the nucleus into the cytoplasm (Figure 4F). Taken together, these data show that SPP1 mediates the nuclear import of ETS2 via MAPK/ERK activation which in turn enhances the transcription of oncogene *JUNB* in HL.

### 3.5. SPP1 Inhibits Apoptosis in HL

**Figure 5:**
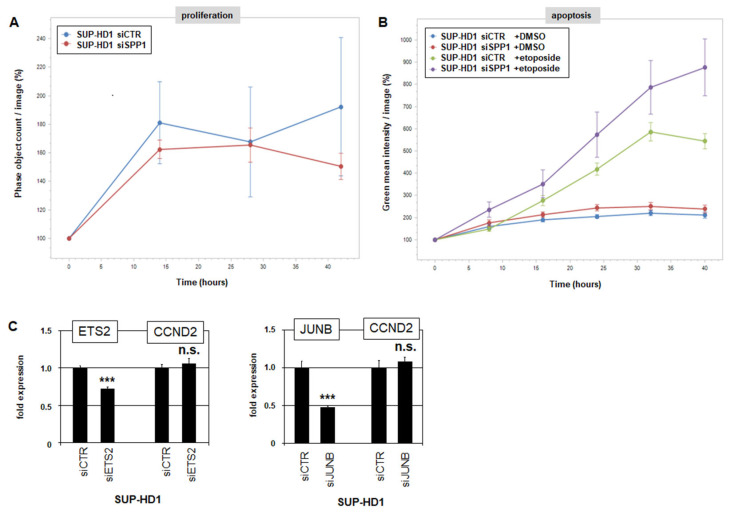
Functional analyses of SPP1 in SUP-HD1 cells. (**A**) Live-cell imaging analysis of proliferating cells treated for siRNA-mediated knockdown of *SPP1*. (**B**) Live-cell imaging analysis of apoptotic cells treated for siRNA-mediated knockdown of *SPP1*. (**C**) RQ-PCR analysis of *CCND2* in SUP-HD1 treated for siRNA-mediated knockdown of *ETS2* (left) and *JUNB* (right). Statistical significance was assessed by Student’s *t*-Test (two-tailed) and the calculated *p*-values are indicated by asterisks (*** *p* < 0.001) or as not significant (n.s.).

Finally, we performed functional analyses of the role of SPP1 in HL cell line SUP-HD1. Their proliferation was quantified after the siRNA-mediated knockdown of *SPP1* using a live-cell imaging system. However, this treatment showed no significant impact (Figure 5A), indicating an absent role of SPP1 in cell proliferation. Next, the cells were additionally treated with apoptosis-inducer etoposide. Live-cell imaging analysis revealed a significant increase in apoptotic cells after SPP1-knockdown as compared to controls (Figure 5B), indicating a role of SPP1 in cell survival. RQ-PCR analysis of SUP-HD1 cells after knockdown of *ETS2* or *JUNB* showed no effect on cyclin D2 (*CCND2*) expression (Figure 5C), supporting the absent role of SPP1-signalling in proliferation.

## 4. Discussion

**Figure 6:**
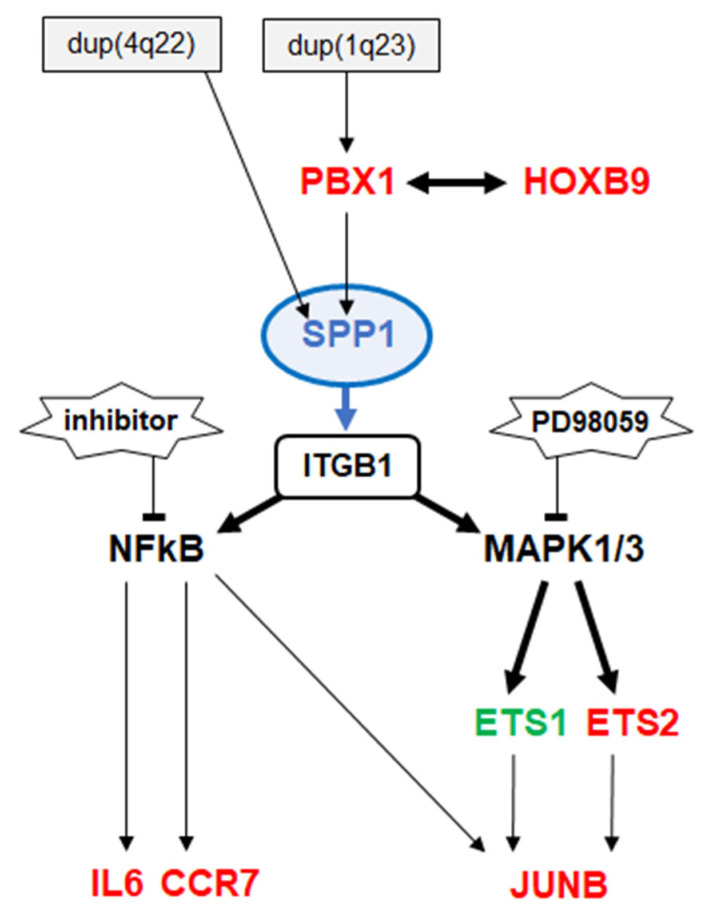
This diagram summarizes the results of the study. Chromosomal aberrations mediate copy number gains of *SPP1* and *PBX1*. HOXB9 and PBX1 are transcriptional activators of *SPP1*. The ligand SPP1 interacts with integrins which in turn mediate the activation of NFkB and MAPK/ERK. *IL6*, *CCR7* and *JUNB* are activated by NFkB, while MAPK1/3 activates ETS1 and ETS2 which translocate into the nucleus and activate *JUNB* transcription.

Aberrant activation of signalling pathways plays a central pathogenic role in many cancers and represents a hallmark of HL [1,11]. These pathways are stimulated in a paracrine or autocrine mode, depending on the source of the ligand [11]. In this study, we investigated the transcriptional deregulation and the pathogenic function of the cytokine SPP1 in HL. Our results are summarized in Figure 6 and discussed below.

Expression analyses of *SPP1* in normal and malignant cells and tissues showed that SPP1 is absent in developing and mature B-cells but is aberrantly activated in subsets of selected B-cell lymphomas, including HL, DLBCL and MCL. These findings indicate the aberrant autocrine activity of SPP1 in these patients. However, *SPP1* is physiologically expressed in the brain. Therefore, in the DLBCL-related malignancy primary central nervous system lymphoma (PCNSL), SPP1 may act in a paracrine mode but may share downstream activities as described for HL in this study [30].

HL cell line SUP-HD1 expresses high amounts of SPP1 at both RNA and protein levels and was chosen to serve as the experimental model in this study. In SUP-HD1, we uncovered two mechanisms underlying aberrant *SPP1* activation. First, the *SPP1* locus was included within a genomic gain at chromosomal position 4q22. This finding fits the general observation that copy number alterations are frequent in HL and contribute to targeted gene deregulation therein [21,22,23,24]. Furthermore, 4q22 is a genomic region of homozygosity in HL patients, underlining its oncogenic significance for this disease [31]. Second, aberrantly expressed homeodomain TFs HOXB9 and PBX1 activated *SPP1* transcription in SUP-HD1. Our data are supported by findings in osteosarcoma and melanoma, showing that HOXB9 and PBX1, respectively, contribute to *SPP1* activation in these tumours [32,33]. Homeodomain TFs show physiological activities in developing and mature immune and blood cells. We have termed the regular patterns of differential TF-subgroups active in particular hematopoietic entities “codes” [34]. Accordingly, the TALE-code describes the physiological expression of eleven TALE-class homeobox genes in early hematopoiesis and lymphopoiesis, including B-cell development [9]. The TALE-class member *PBX1* is active in hematopoietic progenitors but silent in developing B-cells [9,35]. Thus, deregulated PBX1 drives the aberrant expression of *SPP1* in B-cell-derived HL. *HOXB9* was one the first described homeobox genes that is aberrantly expressed in HL [26].

Our data indicated that SPP1 signals via integrins which in turn activate NFkB- and AP1-factors, in addition to MAPK/ERK [12,13]. All these downstream activities represent known pathogenic mechanisms in HL [1]. Here, we showed the SPP1-mediated activation of NFkB and MAPK/ERK via integrin ITGB1, and detailed the subsequent *JUNB* upregulation by ETS factors. However, NFkB-activation may not represent a major downstream effect of SPP1-signalling. SPP1-activated MAPK/ERK mediated the nuclear import of ETS2, which in turn enhanced *JUNB* expression. These factors showed no regulatory role for *CCND2* expression in SUP-HD1 as indicated recently [5]. Consistently, SPP1 supported cell survival but not proliferation in this cell line. Thus, SPP1 drives oncogenic hallmark factors in cHL, including ETS2 and JUNB and partly NFkB [1,5,10]. Of note, *JUNB* performs tumour suppressor activity in NLPHL [36], supporting its regulatory connection with SPP1 restricted to cHL. Finally, MAPK (and partly NFkB) are central components in aberrant SPP1-signalling and may therefore represent potential therapeutic targets in affected patients. Targeting of ETS-factors may also have therapeutic potential in this context when confirmed clinically [37].

## 5. Conclusions

This study showed aberrant autocrine activity of SPP1 in classic HL, and uncovered its pathogenic pathway which discloses targeted therapeutic approaches to improve the treatment of relapsed patients.

## Data Availability

The data presented in this study are openly available as indicated in the text.

## Supplementary Materials

The following supporting information can be downloaded at: https://www.mdpi.com/article/10.3390/biomedicines13030735/s1, Table S1: List of used siRNAs; Figure S1: SPP1 activity in patients and tissues; Figure S2: PBX1 and HOXB9 in HL; Figure S3: CD44 and integrins in HL; Figure S4: Expression and regulation of CCR7 and IL6; Figure S5: Expression and regulation of JUNB.
