# Supplementary material for: Aberrant Expression and Oncogenic Activity of SPP1 in Hodgkin Lymphoma
Source: Biomedicines. 2025 Mar 17;13(3):735. doi: 10.3390/biomedicines13030735 (PMC11940585; doi:10.3390/biomedicines13030735)
Supplement: Supplementary file 1 [file biomedicines-13-00735-s001.zip › biomedicines-3484623-supplementary.pdf]

Table S1: List of used siRNAs

Target gene	Catalogue number	Sequence .
ETS1	Hs_ETS1_3	CCGACGAGTGATGGCACTGAA
ETS2	Hs_ETS2_1	CCCGGTCAAGTTGGTTTCAAA
HOXB9	Hs_HOXB9_2	CCGGTGGGCTAGAAAGTACAA
ITGAV	Hs_ITGAV_2	TAGCATGATGTTACAGGAATA
ITGB1	Hs_ITGB1_5	AAAAGTCTTGGAACAGATCTG
JUNB	Hs_JUNB_5	CCCGACGACCACCATCAGCTA
MAPK3	Hs_MAPK3_6	CCCGTCTAATATATAAATATA
PBX1	Hs_PBX1_3	AGACGGAATTTCAACAAGCAA
SPP1	Hs_SPP1_6	CAGGCTGATTCTGGAAGTTCT

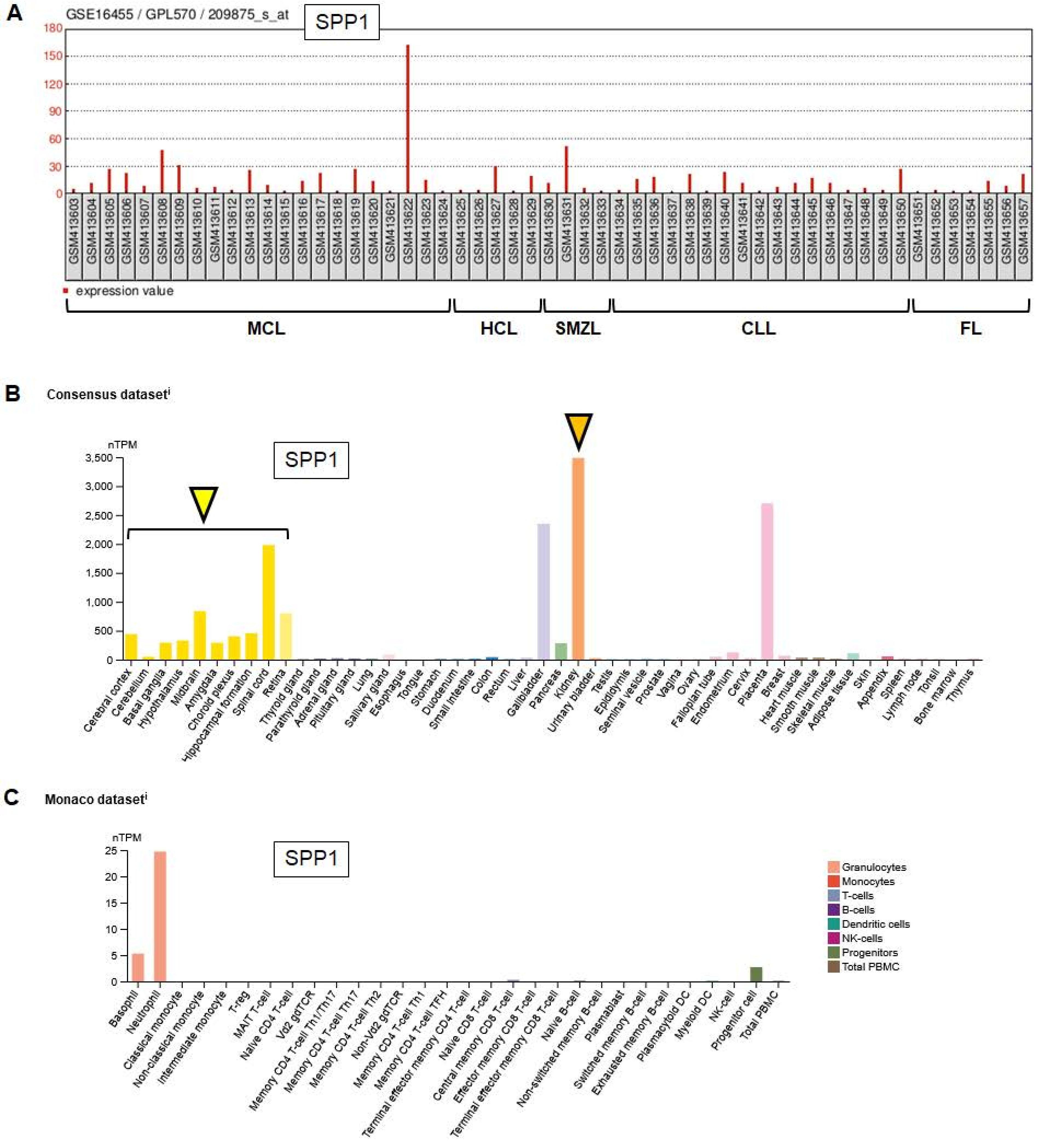

Figure S1: SPP1 activity in patients and tissues. (A) Analysis of public gene expression profiling dataset GSE16455 for SPP1 in selected B-cell malignancies, including mantle cell lymphoma (MCL), hairy cell leukemia (HCL), splenic marginal zone lymphoma (SMZL), chronic lymphoid leukemia (CLL), follicular lymphoma (FL). (B) RNA-seq data analyzed via the Human Protein Atlas from selected tissues, showing SPP1 expression. Arrow heads indicate the brain (yellow) and kidney (orange). (C) RNA-seq data analyzed via the Human Protein Atlas from selected hematopoietic cell types, showing SPP1 expression.

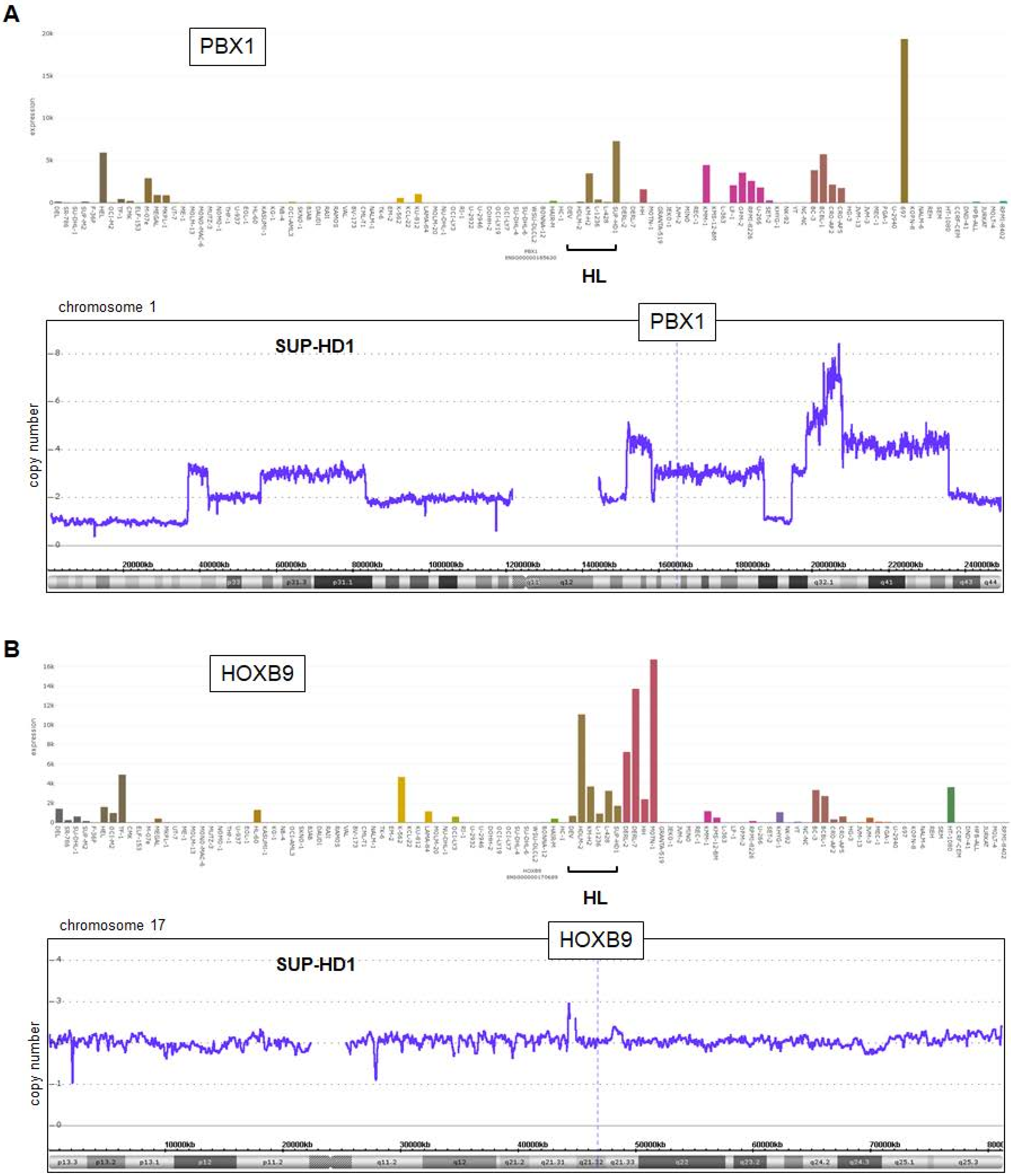

Figure S2: PBX1 and HOXB9 in HL. RNA-seq gene expression data from leukemia/lymphoma cell lines (above) and genomic profiling data from HL cell line SUP-HD1 (below) for (A) PBX1, and (B) HOXB9. Cell lines derived from HL and the according gene loci are indicated, comprising DEV, HDLM-2, KM-H2, L-1236, L-428.

A

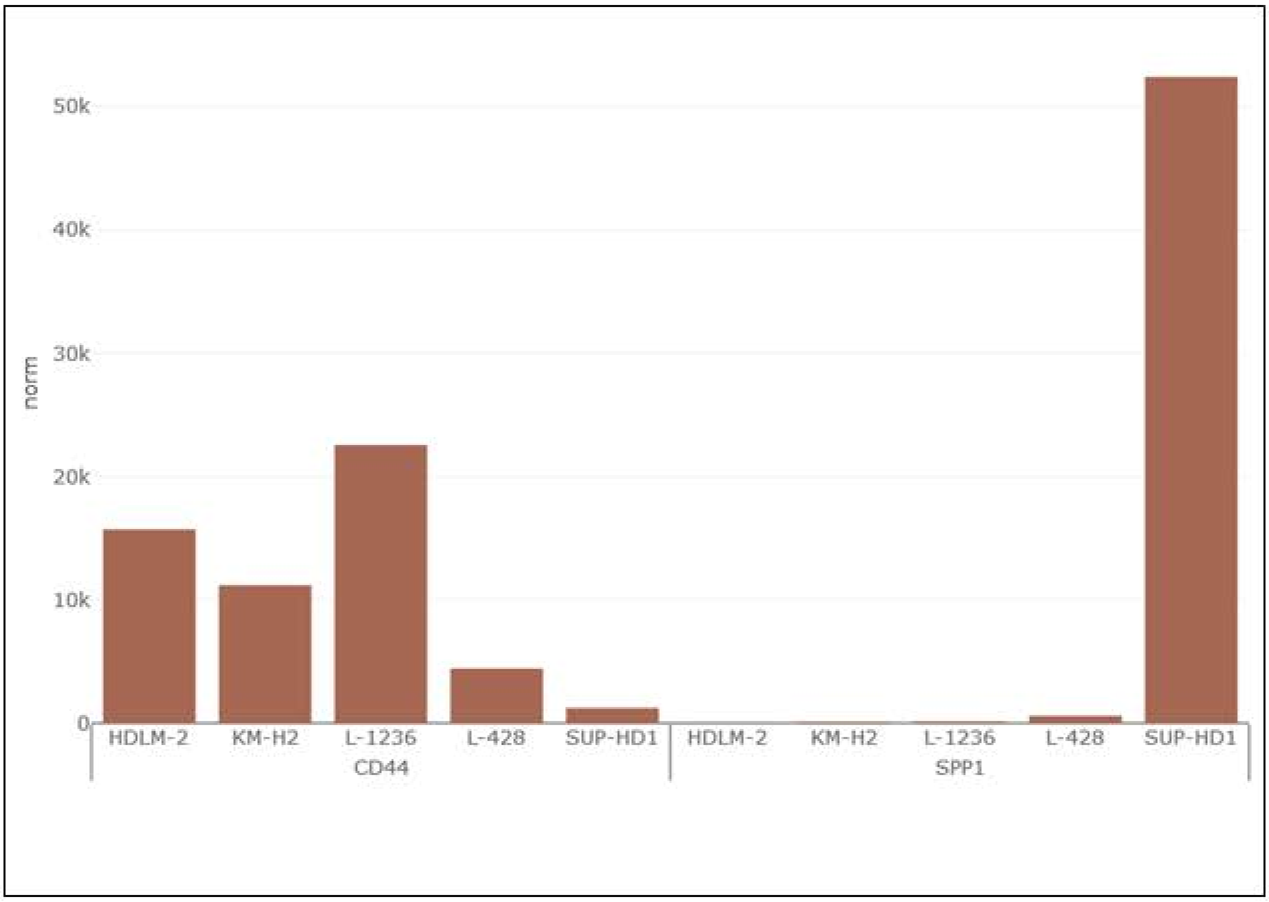

B

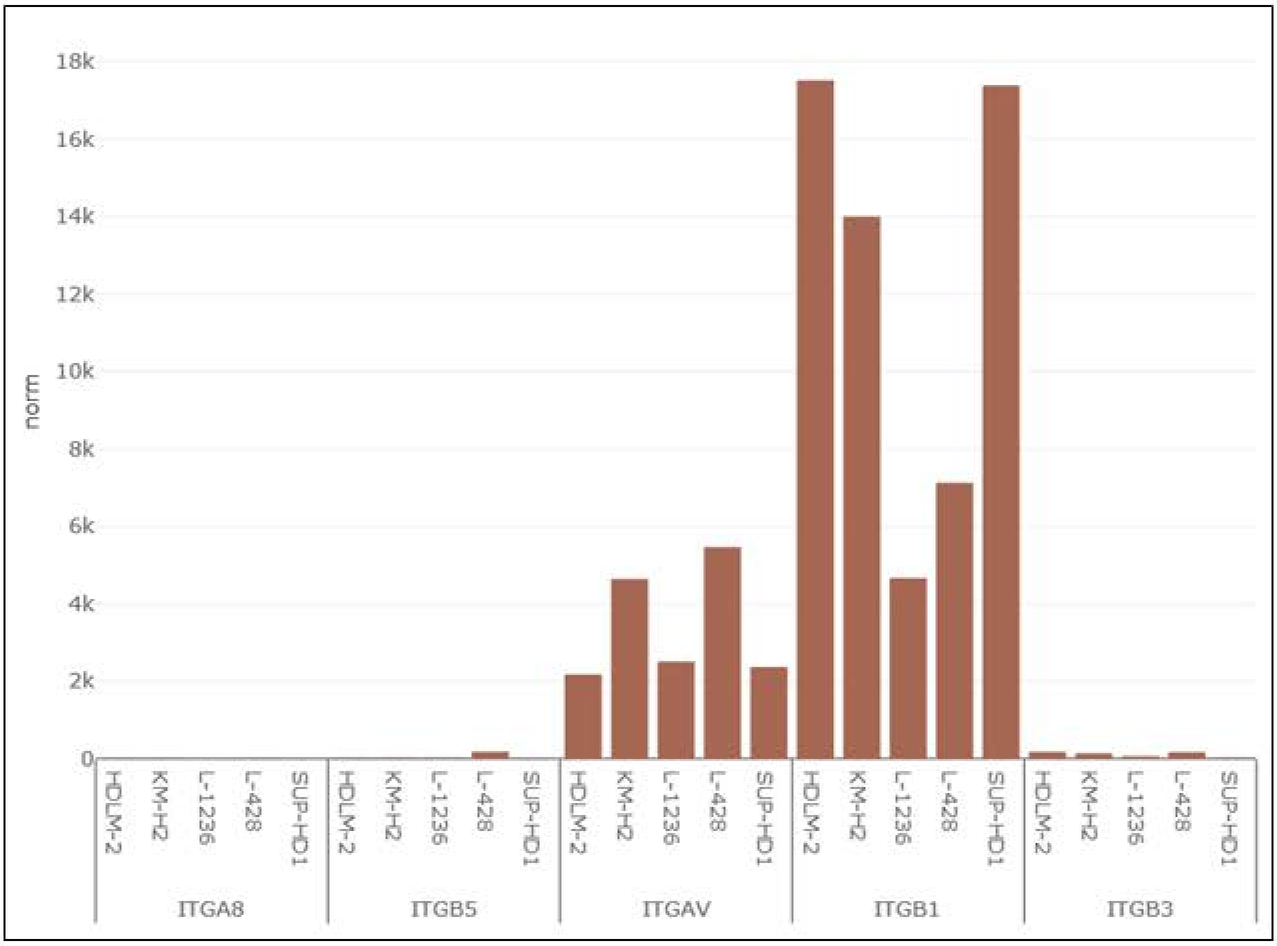

Figure S3: CD44 and integrins in HL. RNA-seq gene expression data from selected HL cell lines for (A) CD44 and SPP1, and (B) the integrins ITGA8, ITGB5, ITGAV, ITGB1 and ITGB3.

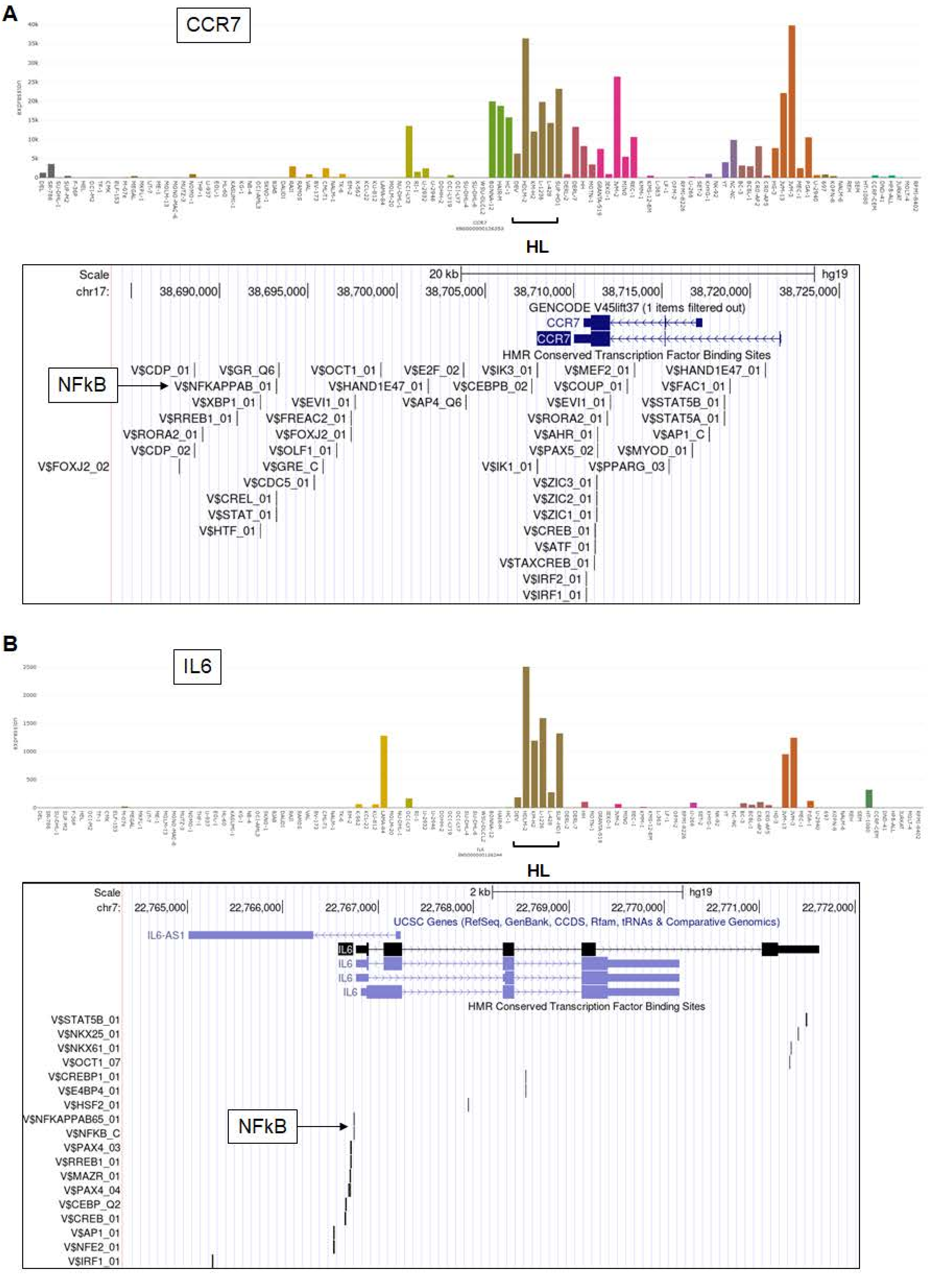

Figure S4: Expression and regulation of CCR7 and IL6. RNA-seq gene expression data from 100 leukemia/lymphoma cell lines (above) and transcription factor binding site analysis using the UCSC genome browser (below) for (A) CCR7, and (B) IL6. Cell lines derived from HL and selected binding sites are indicated, comprising DEV, HDLM-2, KM-H2, L-1236, L-428.

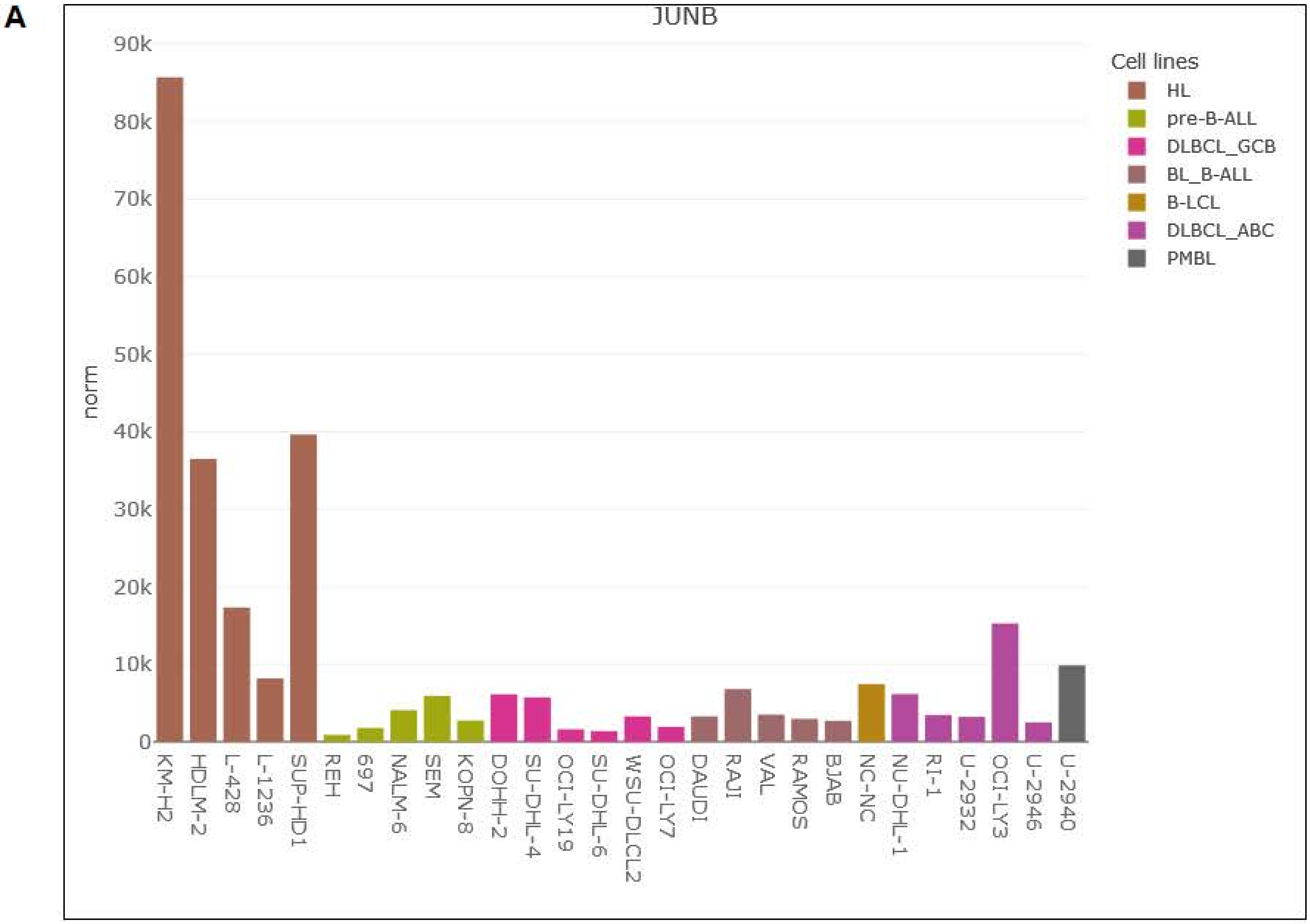

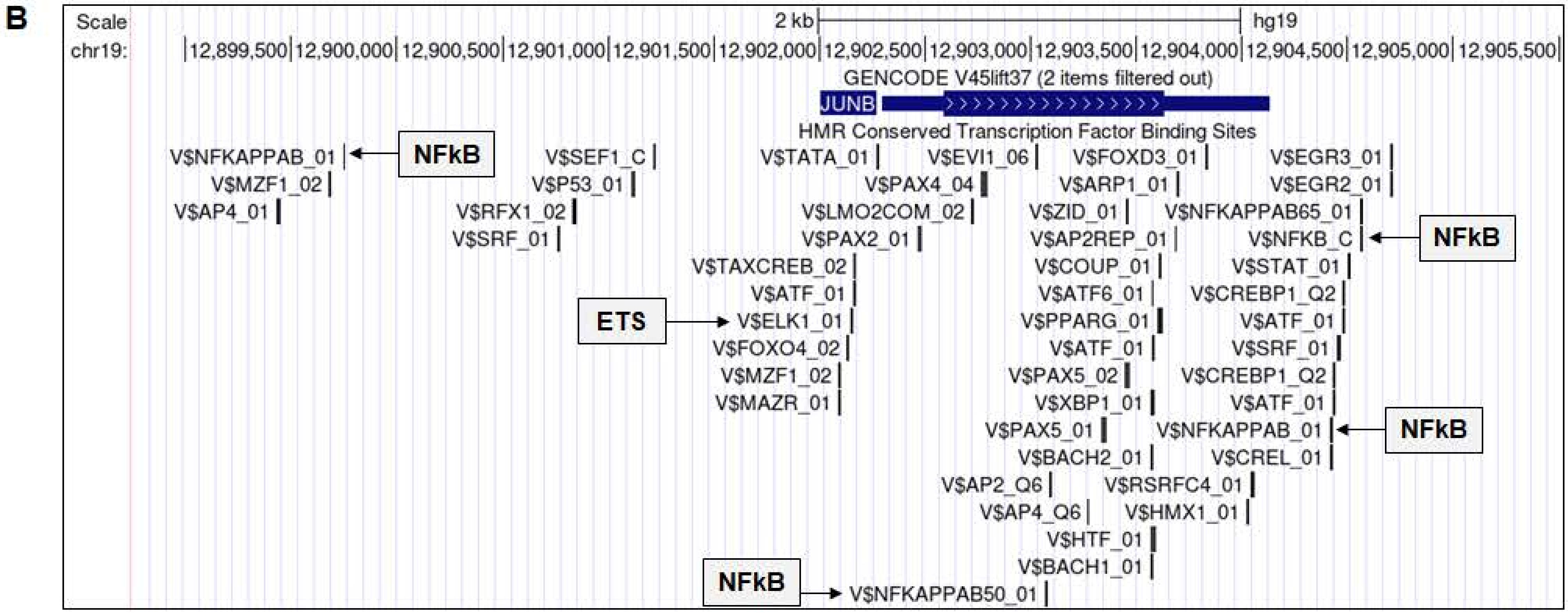

Figure S5: Expression and regulation of JUNB. (A) RNA-seq gene expression data from selected B-cell malignancy derived cell lines (above), and (B) transcription factor binding site analysis using the UCSC genome browser (below) for JUNB. Cell lines derived from HL and selected binding sites are indicated.
